# Twin‐grasper assisted mucosal inverted closure achieves complete healing of large perforations after gastric endoscopic full‐thickness resection

**DOI:** 10.1111/den.14507

**Published:** 2023-02-06

**Authors:** Qinbo Cai, Huafeng Fu, Lele Zhang, Minxuan Shen, Shaoxiong Yi, Rongman Xie, Wentong Lan, Wenqing Dong, Xiaolian Chen, Jie Zhang, Xun Hou, Yulong He, Dongjie Yang

**Affiliations:** ^1^ Center for Gastrointestinal Surgery, The First Affiliated Hospital Sun Yat‐sen University Guangzhou China; ^2^ Laboratory of General Surgery, The First Affiliated Hospital Sun Yat‐sen University Guangzhou China; ^3^ Department of Endoscopy, The First Affiliated Hospital Sun Yat‐sen University Guangzhou China; ^4^ Research Center for Diagnosis and Treatment of Gastric Cancer Sun Yat‐sen University Guangzhou China; ^5^ Silver Snake (Guang Zhou) Medical Technology Co., Ltd Guangzhou China; ^6^ Digestive Medicine Center, The Seventh Affiliated Hospital Sun Yat‐sen University Shenzhen China; ^7^ Guangdong Provincial Key Laboratory of Digestive Cancer Research Shenzhen China

**Keywords:** endoscopic full‐thickness resection, inverted closure, mucosal healing, twin grasper

## Abstract

**Objectives:**

This study aimed to demonstrate the feasibility and safety of a novel twin‐grasper assisted mucosal inverted closure (TAMIC) technique for large perforations after gastric endoscopic full‐thickness resection (EFTR) in a porcine model.

**Methods:**

Iatrogenic large perforations of the stomach were created and closed by an experienced endoscopist using the TAMIC technique in 12 pigs. Repeat gastroscopy was performed in 4 weeks after surgery to examine the wound sites and then the animals were killed. The primary outcomes were the successful TAMIC closure rate and the complete healing rate. Secondary end points included procedure time of TAMIC, complete inverted healing rate, delayed bleeding rate, and postsurgery perforation. Histologies of the wounds were analyzed by hematoxylin–eosin, Masson trichrome, and immunohistochemistry staining.

**Results:**

The median size of the defects was 3.5 (range 2.5–4.5) cm. TAMIC was successfully performed in all the 12 pigs. Complete healing was achieved in 11 pigs 4 weeks after operation as one pig died postsurgery due to severe pneumonia. The median procedure time for TAMIC was 39 (range 23–81) min. The complete inverted healing rate was 45.5% (5/11). No delayed bleeding or postsurgery perforation was observed. Histologic analyses showed that both the epithelium and muscularis mucosae layers were appropriately connected under inverted healing.

**Conclusions:**

Twin‐grasper assisted mucosal inverted closure is feasible and safe for closure of large perforations after gastric EFTR and could be a propagable and promising technique for clinical practice.

## INTRODUCTION

Endoscopic full‐thickness resection (EFTR) is an advanced endoscopic technique that is expected to be a standard technique for resection of gastrointestinal submucosal tumors (SMT) and early gastrointestinal carcinoma with no lymphatic and/or vascular invasion.^1^, ^2^, ^3^ The present indication for EFTR is lesions smaller than 30 mm in diameter and the average diameter of most reports is <20 mm.^4^ One of the reasons is that the main challenge for EFTR is an effective and safe defect closure to prevent peritonitis and delayed perforation, especially closure of large defects with a diameter more than 20 mm. Currently, the most widely accepted techniques mainly use through‐the‐scope clips (TTSC)^5^ or clips combined with endoloops,^6^, ^7^ while defect closure by use of over‐the‐scope clips (OTSC) or endoscopic suturing devices is only reported in a few cases.^4^ However, there are some limitations among the above techniques. Use of TTSCs alone is almost unable to close a defect larger than 20 mm, while high mucosa tension and delayed leakage of gastric juice in the midpoint of wounds after endoloop closure in large defects are significant risk factors for peritonitis and delayed perforation.^6^ Application of OTSC is fundamentally limited by the lesion size and it is not suitable for defects more than 20 mm.^8^ Endoscopic suturing devices, such as overstitch or hand‐suturing, are difficult to handle.^9^, ^10^ In order to simply and effectively close large wounds, we recently developed the twin‐grasper assisted mucosal inverted closure (TAMIC) technique. TAMIC uses the widely accepted TTSC to close large defects after EFTR under the assistance of a twin‐grasper and ensures the muscularis mucosae is closed in inverted and tight apposition. To our knowledge, this study is the first survival study in 12 live pigs to evaluate the feasibility and safety of TAMIC for large gastric perforations.

## METHODS

### Animals and preoperative preparation

This study was approved by the animal experiment ethics committee of Silver Snake (Guang Zhou) Medical Technology Co., Ltd (Guangzhou, China; ss‐2021‐ZSYY). A total of 12 Tibetan pigs (7 months old, weight 21.5–32 kg) were obtained from Songshanhu Mingzhu Experimental Animal Technology (Dongguan, China). Gastric cleaning preparation was performed by providing the animal with a liquid diet for 3 days before the surgery. The liquid diet included 400 g enteral nutritional powder (Ensure; Abbott, Hoofddorp, Netherlands) in 1800 mL water per day, which provided 1800 kcal energy. Anesthesia was induced with 3 mg/kg tiletamine and zolazepam (Zoletil; Virbac, Carros, France) via intramuscular injection, and 2 mg/kg propofol by intravenous injection. Anesthesia was maintained with 1.5% inhaled isoflurane after endotracheal intubation. The animals were ventilated with 8–10 mL/kg of tidal volume at 16–18 respirations per minute and 100% oxygen in a volume‐controlled ventilation model. Cefuroxime (40 mg/kg) was provided to the animal via intravenous drip 30 min before surgery. Physiologic parameters, including heart rate, blood pressure, and blood oxygen saturation, were monitored and maintained in a normal condition throughout the procedure.

### Surgery procedure

**Figure 1:**
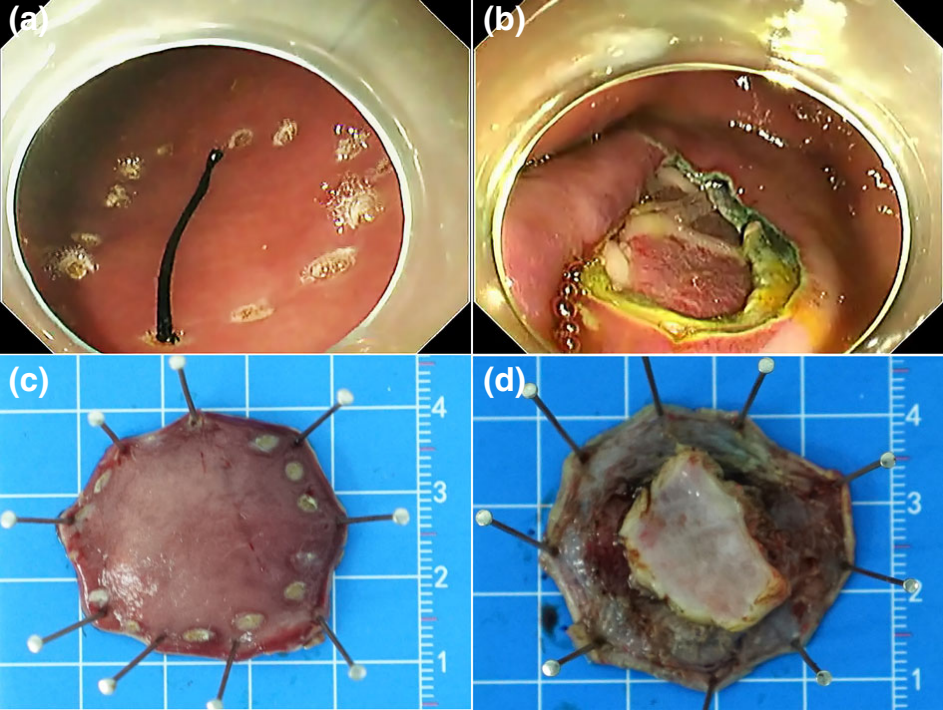
Representative images of the iatrogenic perforation creation. (a) A 2 cm surgical suture was used to ensure the perforations were larger than 2 cm during mucosa labeling. (b) Representative iatrogenic perforation created in this study. (c, d) Representative images of lesions resected.

**Figure 2:**
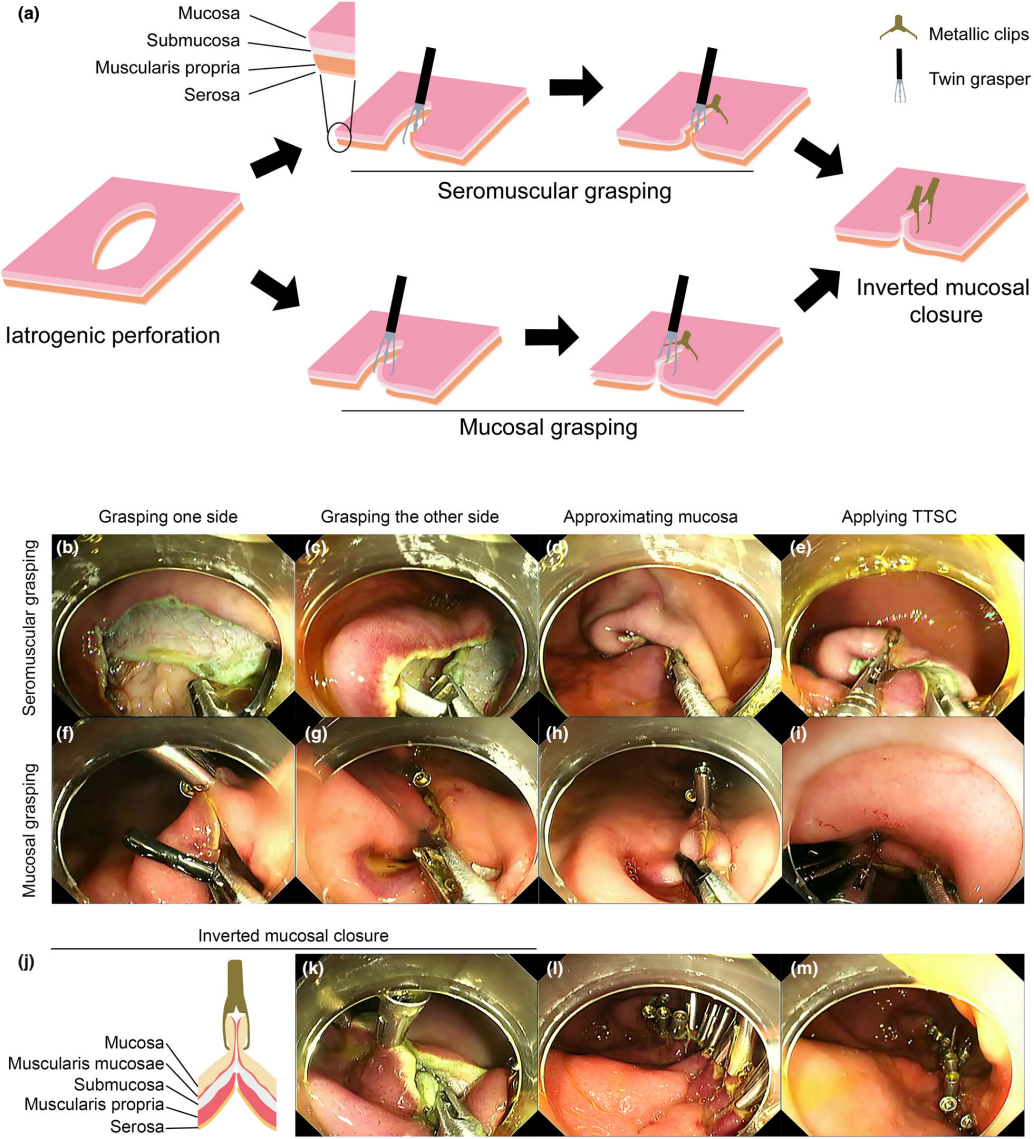
Schema and representative images of the twin‐grasper assisted mucosal inverted closure (TAMIC) technique. (a) Schema of the TAMIC technique. Briefly, the two arms of a twin grasper were opened in turn to grasp each side of the seromuscular or mucosal layer of the perforation, causing the mucosal layer approximated. Through‐the‐scope clips were then sent into another working channel to close the mucosa. (b–e) Representative images of seromuscular grasping. (f–i) Representative images of mucosal grasping. (j) Schema of the inverted mucosal closure. (k) A representative image of inverted mucosal closure. The muscularis mucosae was put in inverted and tight apposition and the clips clamped as much mucosa as possible. (l, m) Representative images for overview of the defects closed by TAMIC.

The EFTR techniques were performed as previously reported.^8^ All the iatrogenic perforations were created in the posterior wall, because the posterior wall is more fixed as part of the lesser omental bursa, which causes higher tension during closure compared to the anterior wall. We believe TAMIC could also be successful in the anterior wall if we could get positive results in the posterior wall. During mucosal labeling, a 2 cm surgical suture was used as a ruler to ensure the perforations were larger than 2 cm (Fig. 1). We decided to create perforations with a diameter slightly larger than 2 cm, because most of the SMTs treated with endoscopy are smaller than 2 cm in clinical practice.^5^, ^7^ For TAMIC, an endoscope with two working channels (Olympus, Tokyo, Japan) was used and arms of a twin grasper (Ovesco, Tuebingen, Germany) were opened in turn to grasp each side of the seromuscular or mucosal layer of the perforation, causing the mucosal layer approximated tightly. Metallic clips (Micro‐Tech, Nanjing, China) were sent into another working channel to close the mucosa (Fig. 2, Videos S1, S2). In this way, the muscularis mucosae was put in inverted and tight apposition (Fig. 2j,k). The large defects were closed from the distant end point to the adjacent one to avoid high tension in the midpoint and to guarantee good operator visibility. A 20G needle was inserted in the right upper quadrant to relieve the pneumoperitoneum after the artificial perforation was created.

### Post‐EFTR management and assessments

Pigs were fasted on the day of surgery for 24 h. Then a half volume liquid diet (200 mL every 3 h, total 1000 mL daily) was provided for another 2 days. At the same time, animals were provided with intravenous nutrition and fluids (400 mL multiple electrolytes, 250 mL fat emulsion injection, 150 mL 5% glucose, and 200 mL 0.9% sodium chloride injection). A normal volume liquid diet (400 mL every 3 h, total 2000 mL daily) was applied from day 3 to day 7 without intravenous fluids. After that, animals were fed with a general diet. Cefuroxime (40 mg/kg/day) was provided until day 3. Repeat gastroscopy was performed in 4 weeks after surgery to examine the wound sites and then the animals were sacrificed. The primary outcomes were the successful TAMIC closure rate and the complete healing rate. Secondary end points included procedure time of TAMIC, complete inverted healing rate, delayed bleeding rates, and postsurgery perforation. Complete inverted healing was defined as complete healing of both the mucosa and muscularis mucosae layers, without ulcer. Delayed bleeding was defined as melena or intra‐abdominal bleeding, which is diagnosed with nonnormal physiologic parameters, hemorrhagic abdominal effusion under ultrasound examination, and abdominal puncture. Postsurgery perforation was preliminarily judged according to reduced intake, fever, increased abdominal protuberance, and muscular tension. We defined the clip closure condition as satisfactory condition when the clips clamp enough mucosal tissue and the depth of the two sides are almost equal (Fig. S1). The definition of the whole closure was as follows: very good, over 90% of the clips are applied in a satisfactory condition; good, 70–90% of the clips are applied in a satisfactory condition; and bad, <70% of the clips are applied in a satisfactory condition.

### Statistical analysis

Statistical analyses were performed using GraphPad Prism (version 9; GraphPad Software, San Diego, CA, USA). Mann–Whitney *U*‐test was used to compare the results of lesion size, closure time, and number of clips, while Fisher's exact test was used to compare categorical variables. Two‐tailed tests and an *α* of 0.05 were used for all statistical analyses.

## RESULTS

The median body weight of the 12 pigs involved in this study was 27.0 (range 21.5–32.0) kg. EFTR was successfully performed in all the animals and the median long and short diameters of the wounds were 3.5 (range 2.5–4.5) cm and 3.2 (range 2.5–4.0) cm, respectively. All the 12 defects were created in the posterior gastric wall, including 1 (8.3%) in the antrum, 8 (66.7%) in the body, and 3 (25.0%) in the junction between antrum and body. TAMIC successfully closed all the 12 perforations and the median procedure time for TAMIC was 39 (range 23–81) min. 83.3% (10/12) of the defects were closed in a direction vertical to the major axis of the stomach while others were performed in a parallel way. 41.7% (5/12) of the wounds were closed in a very good condition and the median number of clips used per perforation was 14 (range 11–19) (Table 1).

**Table 1:** Lesion characteristics and technique outcomes (*n* = 12)

Characteristics/outcomes	Proportion (%, *n*/*N*)/median (range)	Mean ± SD
Weight, kg	27.0 (21.5–32.0)	26.8 ± 3.6
Lesion size, cm		
Long diameter	3.5 (2.5–4.5)	3.6 ± 0.5
Short diameter	3.2 (2.5–4.0)	3.3 ± 0.4
Lesion location		
Antrum	8.3% (1/12)	NA
Body	66.7% (8/12)	NA
Junction between antrum and body	25.0% (3/12)	NA
Complete closure rate	100% (12/12)	NA
Procedure time of TAMIC, min	39 (23–81)	42.8 ± 17.4
Closure direction		
Parallel to major axis of stomach	16.7% (2/12)	NA
Vertical to major axis of stomach	83.3% (10/12)	NA
Satisfaction of closure		
Good	58.3% (7/12)	NA
Very good	41.7% (5/12)	NA
Number of clips per perforation	14 (11–19)	14.5 ± 2.5

NA, not available; SD, standard deviation; TAMIC, twin‐grasper assisted mucosal inverted closure.

**Figure 3:**
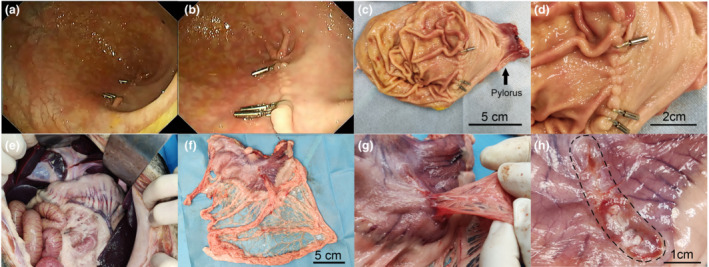
Representative images of complete inverted healing. (a) Overview of the stomach cavity without apparent deformation. (b–d) Representative images of the wound site. (e) Abdominal cavity with no adhesion. (f–h) Representative images of the wound site in seromuscular layer.

**Figure 4:**
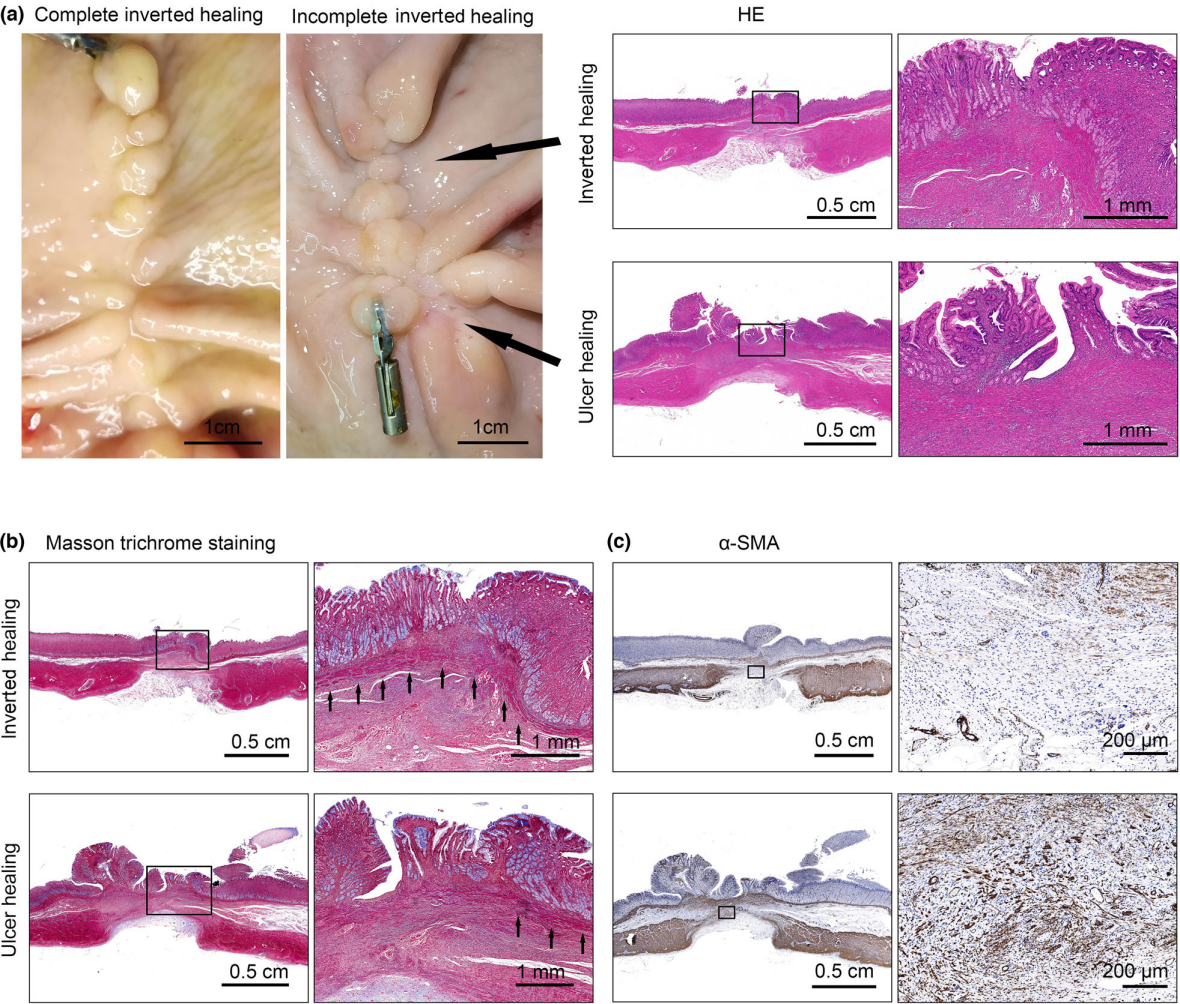
Anatomic differences between inverted healing and ulcer healing. (a) Left panel: representative images of complete and incomplete inverted healing. Right panel: hematoxylin–eosin (HE) staining of the inverted healing sites and the ulcer healing sites. (b) Masson trichrome staining of the inverted healing sites and the ulcer healing sites. Black arrows indicate the muscularis mucosae layer. (c) Immunohistochemistry staining of alpha‐smooth muscle actin (α‐SMA).

Two of the 12 pigs developed pneumonia after surgery (confirmed by chest X‐ray, might be ventilation‐associated infection), and one of them recovered after antibiotic therapy for 1 week. Another died 3 weeks after surgery and autopsy confirmed severe pneumonia while there was no ascites or perforation in the stomach (Fig. S2). This animal was excluded from the final analysis of postsurgery outcomes due to its early death. So a total of 11 animals were included in the postsurgery outcome analyses. All the wounds were completely healed (Fig. 3, Table 2). However, only 45.5% (5/11) of them had expected healing appearance, in which the mucosa was healed in an inverted way without any ulcer healing (defined as complete inverted healing, Fig. 4a), and others contained part ulcer healing (defined as incomplete inverted healing, Fig. 4a). Compared to ulcer healing, there was a closer to normal epithelium structure in the inverted healing site (Fig. 4a). The muscularis mucosae layer was appropriately connected under inverted healing, while there was no muscularis mucosae in the ulcer healing sites (Fig. 4b). In addition, the degree of neovascularity and fibroblasts in the submucosa was smaller in the inverted healing sites than that in the ulcer healing groups (Fig. 4c). There was no delayed bleeding or postsurgery perforation observed. We also found that there was only one stomach showing deformity inside, while there was no deformity outside the stomach. What is more, the wounds in seromuscular layers of all the animals were not closed by TAMIC, but were replaced with scar (Figs 3h,4), and the median long and short parameters of the seromuscular wounds were 2.5 (range 1.4–3.5) cm and 1.5 (range 0.9–2.5) cm, respectively. There were adhesions with omentum majus in all the wounds while no other intraperitoneum adhesion was found. Interestingly, clarified yellow ascites (about 100 mL) was found in one of the animals without any evidence of perforation (Table 2).

**Table 2:** Postsurgery outcomes (*n* = 11)

Outcomes	Proportion (%, *n*/*N*)/median (range)	Mean ± SD
Weight gained, kg	1.5 (−3.0–6.0)	1.5 ± 2.6
Clips remained per animal	1 (0–4)	1.2 ± 1.5
Complete healing rate	100% (11/11)	NA
Complete inverted healing rate	45.5% (5/11)	NA
Delayed bleeding	0.0% (0/11)	NA
Postsurgery perforation	0.0% (0/11)	NA
Wound in seromuscular layer, cm		
Long parameter	2.5 (1.4–3.5)	2.6 ± 0.5
Short parameter	1.5 (0.9–2.5)	1.5 ± 0.4
Deformation inside	9.1% (1/11)	NA
Deformation outside	0.0% (0/11)	NA
Adhesion with omentum majus	100% (11/11)	NA
Intraperitoneum adhesion	0.0% (0/11)	NA
Ascites	9.1% (1/11)	NA

NA, not available; SD, standard deviation.

We were wondering if there was any risk factor for incomplete inverted healing or ulcer healing. However, the results showed that there was no statistical significance between the incomplete and complete inverted healing groups among possible risk factors, such as lesion size, lesion location, closure time, or condition of satisfaction of closure (Table 3).

**Table 3:** Risk factors for incomplete inverted healing

Factors	Incomplete (*n* = 6)	Complete (*n* = 5)	*P*‐value
Lesion size, mm, median (range)/mean ± SD	3.6 (3.2–4.3)/3.7 ± 0.5	3.5 (2.5–3.8)/3.3 ± 0.5	0.41
Lesion location, *n* (%)			0.63
Antrum	1 (16.7)	0 (0.0)	
Body	4 (66.7)	4 (80.0)	
Junction between antrum and body	1 (16.7)	1 (20.0)	
Closure time, min, median (range)/mean ± SD	66.5 (30.0–84.0)/59.2 ± 20.1	36.0 (31.0–56.0)/42.4 ± 12.5	0.16
Closure direction, *n* (%)			0.45
Parallel to major axis of stomach	2 (33.3)	0 (0.0)	
Vertical to major axis of stomach	4 (66.7)	5 (100.0)	
Satisfaction of closure, *n* (%)			0.24
Good	5 (83.3)	2 (40.0)	
Very good	1 (16.7)	3 (60.0)	
Number of clips, median (range)/mean ± SD	15.0 (12.0–19.0)/15.7 ± 2.8	13.0 (11.0–14.0)/12.8 ± 1.3	0.06

SD, standard deviation.

## DISCUSSION

This study demonstrated that TAMIC could effectively close large gastric perforation with a 100% complete healing rate. Unlike traditional TTSC technique, TAMIC used a double‐channel endoscope and a twin‐grasper to approximate the two sides of the defect before applying the TTSC to close the wounds in an inverted way. Technically, TAMIC could close defects regardless of their size, although the longest diameter was 4.5 cm in this study. This is a great improvement as traditional TTSC could hardly close defects with a diameter longer than the width of the open clips.^4^, ^5^, ^11^ In addition, this study indicated that TAMIC was very safe as no delayed bleeding or perforation was observed in all the animals. These results are very inspiring because the porcine gastric mucosa is excessively thick and the dietary control was much harder than that in human.^10^ The inspiring feasibility and safety in porcine study are strong indications for further clinical practice in human. In this study, the median closure time for TAMIC was 39 (range 23–81) min, which was comparable with endoloops (8–20 min for smaller defects, range 0.4–3 cm),^6^ but little shorter than suturing techniques. For example, the double‐armed bar suturing system took a median time of 77 min to close defects with a median size of 45 mm.^12^ Since TAMIC was a novel technique, more practice and summary of the technique tips might be helpful to shorten the procedure time.

When closing the defects, TAMIC ensured the muscularis mucosae was put in inverted and tight apposition and the TTSC could clamp as much mucosa as possible (Fig. 2). These two strengths prevented the mucosa from slipping and provided better condition for mucosal healing, especially the continuity of the muscularis mucosae, which was disappeared in the ulcer healing (Fig. 4) or when the mucosa epithelial, rather than the muscularis mucosae, were put in apposition by endoloops,^13^ O‐ring,^14^ Loop 9,^15^ or reopenable clip over line method.^16^, ^17^ So, it is quite important to adjust the TTSC and make sure both sides of the mucosa are in an inverted apposition. The degree of neovascularity and fibroblasts in the submucosa was also found smaller in the inverted healing sites than that in the ulcer healing groups, which is similar with results in hand‐suturing closure.^18^ For clinical advantages, we found that all the mucosal layers healed well in the complete inverted healing group, while the mucosal layer was still partly covered by granulation tissue in the incomplete group. This means patients with complete inverted healing might have a shorter recovery time compared to those with uninverted healing, which might be important for patients with a high risk of delayed bleeding, such as those taking anticoagulants.^19^ In addition, there was less granulation tissue in the complete group, which meant less inflammation. Last, the complete groups had normal structure of mucosal epithelium and a continuous muscular mucosae, which might be a guarantee for gastric function.

OTSC^20^ and the grasp‐and‐loop closure (GAL)^21^ are another two methods that can close the mucosal layers in an inverted way, while the overstitch is the only reported endoscopic technique that can ensure closure of the full gastric wall.^22^ TAMIC is performed under a double‐channel endoscope, which is also required for the GAL and the interrupted endoloop suture.^6^, ^21^ The through‐the‐scope twin clips might clamp less mucosa than TAMIC does, since there is no device to reduce tension during closure (Table S1).^23^

Inverted healing was supposed to be the best healing condition in this study. However, only 45.5% (5/11) of the defects achieved complete inverted healing without any ulcer healing. Ulcer healing is supposed to be caused by the early detaching of the TTSC. We then explored the possible risk factors of incomplete inverted healing or ulcer healing (Table 3). Although there was no significant statistical difference among all the possible risk factors, the complete inverted healing group required less closure time (median procedure time: 36.0 min vs. 66.5 min), and had a higher proportion of very good closure condition (60.0% vs. 16.7%) and less parallel closure direction (0.0% vs. 33.3%) compared with the incomplete inverted healing group. These results indicated that the complete inverted healing could be improved by an experienced endoscopist with enough practice of TAMIC. Also, modification of the clips to provide stronger closure effect should be helpful.

Two grasping techniques, the seromuscular grasping and mucosal grasping (Fig. 2), were used to approximate perforation margins. Although we used the seromuscular and mucosal grasping methods in a hybrid manner in most cases, we divided them into two groups according to which method was mainly used (over 70% clips). It seems that there is no significant difference in outcomes between the two groups (Table S2). However, we would like to recommend seromuscular grasping in the first half of closure according to our experience, since there is great tension and mucosal grasping might cause mucosal damage. While mucosal grasping is easy to be performed in the last half of closure and works perfectly to keep mucosa inverted.

There are some limitations in this study. First, although the anatomic structure of porcine stomach is similar to that of humans, we found that the porcine stomach wall was much thicker than that of humans, and the gastrointestinal motility might be different. Thus, studies in live pigs might not completely simulate the procedure in humans. Second, this study was a single‐arm study, so there were no comparison results with other EFTR closure techniques, such as endoloop and suturing. Third, all the wounds in this study were created in the posterior wall of the stomach, further studies are needed to elucidate the effectiveness of TAMIC in other sites of the gastric wall. TAMIC could theoretically be applied to other anatomical parts of the stomach or other alimental organs, such as duodenum, colon, and rectum. However, it may be difficult to apply TAMIC in the fundus or the lesser curvature, as it is hard to adjust the twin‐gasper and the metal clip in a proper direction. As for perforations larger than 4.5 cm, it is better to combine TAMIC with techniques, such as overstitch and OTSC, because they can strongly close the full thickness of the gastric wall and reduce tension.^20^, ^22^

In conclusion, results of this study demonstrate that TAMIC is feasible and safe for closure of large gastric perforations. Although further clinical studies in human are required, TAMIC is supposed to be a propagable and promising technique for full‐thickness wall defect closure under flexible endoscopes.

## CONFLICT OF INTEREST

Authors declare no conflict of interest for this article.

## FUNDING INFORMATION

This work was supported by the National Natural Science Foundation of China (82172637), the Non‐profit Central Research Institute Fund of Chinese Academy of Medical Sciences (2021‐JKCS‐004), the Guangdong Provincial Key Laboratory of Digestive Cancer Research (2021B1212040006), the GuangDong Basic and Applied Basic Research Foundation (2021A1515110495), and the China Postdoctoral Science Foundation (2022M713583, 2022T150756).

## Supporting information


**Figure S1** Representative images of satisfactory condition of closure.
**Figure S2** Images about the animal died of pneumonia without abdominal complications. (a) No adhesion was found in the abdominal cavity. (b) No ascites was found. (c, d) The seromuscular defect was totally covered and stuck by the omentum majus. (e) Overview of the lungs. (f, g) Hematoxylin–eosin staining showed severe pneumonia of almost all the lungs. Red arrow, the seromuscular wound site.
**Table S1** Tools and techniques for closing perforations after endoscopic full‐thickness resection in stomach^#^.
**Table S2** Outcomes between the mucosal grasping and seromuscular grasping groups.

**Video S1** Representative case of twin‐grasper assisted mucosal inverted closure using seromuscular grasping.

**Video S2** Representative case of twin‐grasper assisted mucosal inverted closure using mucosal grasping.
