# Supplementary material for: Twin‐grasper assisted mucosal inverted closure achieves complete healing of large perforations after gastric endoscopic full‐thickness resection
Source: Dig Endosc. 2023 Feb 6;35(6):736–44. doi: 10.1111/den.14507 (PMC12136266; doi:10.1111/den.14507)
Supplement: Supplementary file 1 — Figure S1 Representative images of satisfactory condition of closure. Figure S2 Images about the animal died of pneumonia without abdominal complications. (a) No adhesion was found in the abdominal cavity. (b) No ascites was found. (c, d) The seromuscular defect was totally covered and stuck by the omentum majus. (e) Overview of the lungs. (f, g) Hematoxylin–eosin staining showed severe pneumonia of almost all the lungs. Red arrow, the seromuscular wound site. Table S1 Tools and techniques for closing perforations after endoscopic full‐thickness resection in stomach#. Table S2 Outcomes between the mucosal grasping and seromuscular grasping groups. [file DEN-35-736-s002.pdf]

Supplementary figures

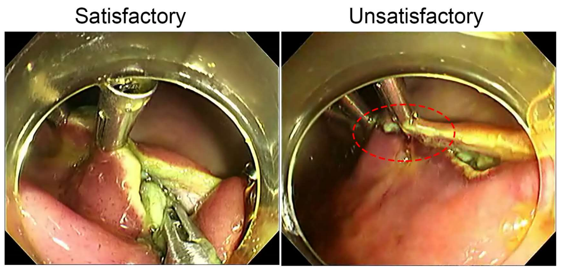

Figure S1 Representative images of satisfactory condition of closure.

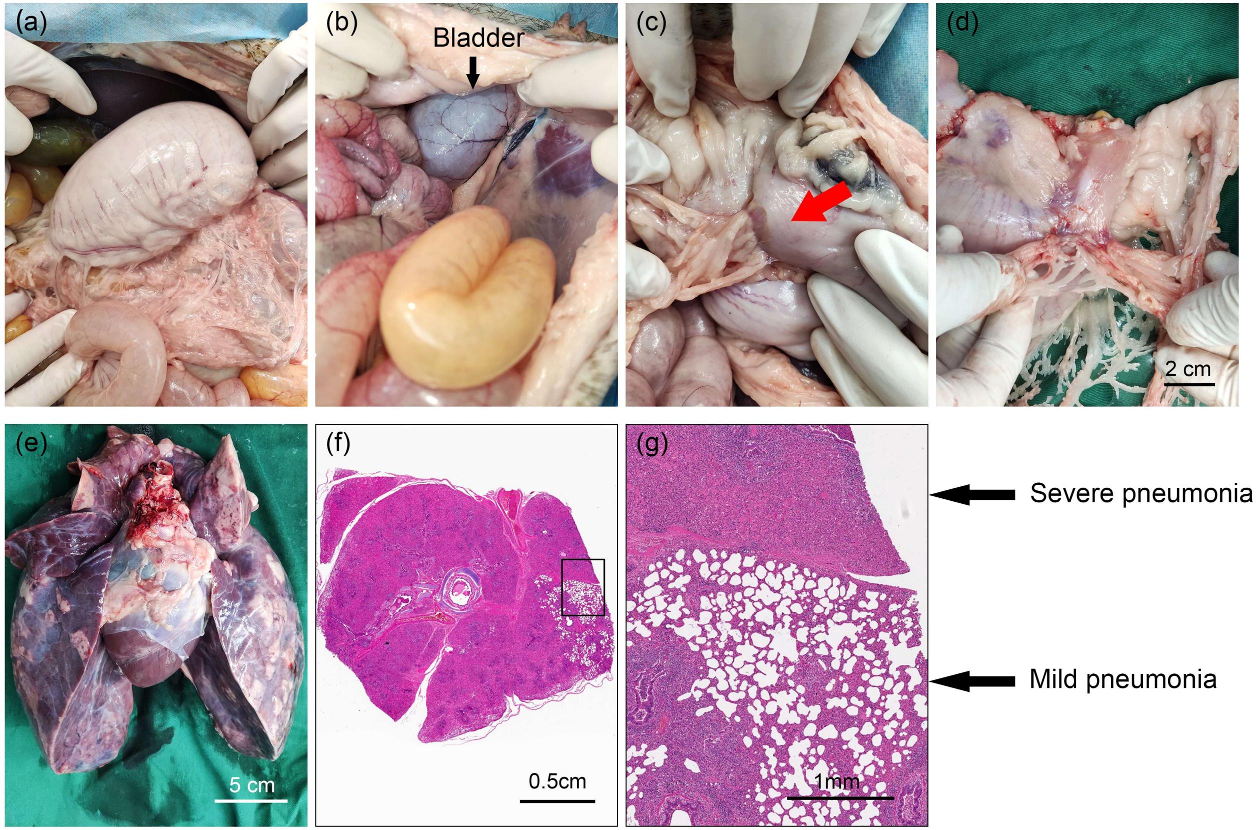

Figure S2 Images about the animal died of pneumonia without abdominal complications. (a) No adhesion was found in the abdominal cavity. (b) No ascites was found. (c) and (d) The seromuscular defect was totally covered and stuck by the omentum majus. (e) Overview of the lungs. (f) and (g) HE staining showed severe pneumonia of almost all the lungs. Red arrow: the seromuscular wound site.

Supplementary tables

Table S1 Outcomes between the mucosal grasping and seromuscular grasping groups.

Outcomes	Seromuscular grasping (n = 7)	Mucosal grasping (n = 4)	*P* value
Successful closure rate/proportion (%, n/N)	100.0% (7/7)	100.0% (4/4)	ns
Closure time/median (range)	35.5 (23.0 - 81.0)	44.5 (33.0 - 55.0)	ns
Clips remained per animal/median (range)	1.0 (0.0 - 4.0)	0.5 (0.0 - 1.0)	ns
Complete healing rate/proportion (%, n/N)	100.0% (7/7)	100.0% (4/4)	ns
Complete inverted healing rate/proportion (%, n/N)	28.6% (2/7)	75.0% (3/4)	ns
Delayed bleeding/proportion (%, n/N)	0.0% (0/7)	0.0% (0/4)	ns
Post-surgery perforation/proportion (%, n/N)	0.0% (0/7)	0.0% (0/4)	ns
Wound in seromuscular layer, cm			
Long parameter/median (range)	2.5 (1.4 - 3.1)	2.9 (2.5 - 3.5)	ns
Short parameter/median (range)	1.5 (0.9 - 1.6)	1.7 (1.1 - 2.5)	ns
Deformation inside/proportion (%, n/N)	14.3% (1/7)	0.0% (0/4)	ns
Deformation outside/proportion (%, n/N)	0.0% (0/7)	0.0% (0/4)	ns
Adhesion with omentum majus/proportion (%, n/N)	100.0% (7/7)	100% (4/4)	ns
Intraperitoneum adhesion/proportion (%, n/N)	0.0% (0/7)	0.0% (0/4)	ns
Ascites/proportion (%, n/N)	14.3% (1/7)	0.0% (0/4)	ns

ns: not significant.

Table S2 Tools and techniques for closing perforations after EFTR in stomach#.

Tools or techniques	Devices used	Effectors	Closed layers	Reinsertion	Applicable perforation size	Applicability to EFTR	Inverted closing	Closure cost
TTSC[1, 2]	Clips and one-channel endoscopes	Clips	Mucosa	No	Small	Weakly closed	No	Low
OTSC[3]	OTSC, twin-Graspers and one-channel endoscopes	OTSC	Mucosa and submucosa, partially muscular layer and serosa	Yes	Small	Strongly closed	Yes	High
Overstitch[4, 5]	Overstitch system	Suture	Full gastric wall	No	Large	Strongly closed	No	High
Endoloops and TTSC[6-8]	Clips, endoloops and one/double-channel endoscopes	Clips and endoloops	Mucosa and partially submucosa	No	Moderate	Weakly closed	No	Low
O-ring/Loop 9 and TTSC[9, 10]	Clips, O-ring/Loop and one-channel endoscopes	Clips	Mucosa and partially submucosa	Yes	Large	Weakly closed	No	Low
TTS-TCs[11]	TTS-TCs, clips and one-channel endoscopes	TTS-TCs and clips	Mucosa and partially submucosa	No	Large	Weakly closed	No	Low
ROLM[12, 13]	Clips, nylon line and one-channel endoscopes	Clips and line	Mucosa and partially submucosa	No	Large	Moderate closed	No	Low
GAL[14]	Endoloop, Grasper and double-channel endoscopes	Endoloop	Mucosa and submucosa	No	small	Moderate closed	Yes	Low
TAMIC	Clips, twin-graspers and double-channel scope	Clips	Mucosa and submucosa	No	Large	Moderate closed	Yes	Low

#: We only included tools or techniques with animal or patient data to approve the successful wound healing. TTSC: through the scope clips; OTSC: over the scope clips; TTS-TCs: through-the-scope twin clips; ROLM: reopenable clip over line method; GAL: grasp-and-loop closure; TAMIC: twin-grasper assisted mucosal inverted closure.

Reference

1 Zhao Y, Pang T, Zhang B *et al.* Retrospective comparison of endoscopic full-thickness versus laparoscopic or surgical resection of small (≤ 5 cm) gastric gastrointestinal stromal tumors. *J Gastrointest Surg* 2020; **24**: 2714-21.

2 Zhou PH, Yao LQ, Qin XY *et al.* Endoscopic full-thickness resection without laparoscopic assistance for gastric submucosal tumors originated from the muscularis propria. *Surg Endosc* 2011; **25**: 2926-31.

3 Guo J, Liu Z, Sun S *et al.* Endoscopic full-thickness resection with defect closure using an over-the-scope clip for gastric subepithelial tumors originating from the muscularis propria. *Surg Endosc* 2015; **29**: 3356-62.

4 Azzolini F, Cecinato P, Iori V, De Marco L, Sassatelli R. Endoscopic full-thickness resection for suspected residual rectal neuroendocrine tumor and closure of the defect with a new suturing system. *Endoscopy* 2015; **47 (Suppl 1)**: E556-7.

5 Jovani M, Zhang L, Huang Y, Kumbhari V. Multi-layer endoscopic suturing: A novel method of gastric fistula closure. *Endosc Int Open* 2021; **9**: E1520-e3.

6 Shi D, Li R, Chen W *et al.* Application of novel endoloops to close the defects resulted from endoscopic full-thickness resection with single-channel gastroscope: A multicenter study. *Surg Endosc* 2017; **31**: 837-42.

7 Shi Q, Chen T, Zhong YS *et al.* Complete closure of large gastric defects after endoscopic full-thickness resection, using endoloop and metallic clip interrupted suture. *Endoscopy* 2013; **45**: 329-34.

8 Ye LP, Yu Z, Mao XL, Zhu LH, Zhou XB. Endoscopic full-thickness resection with defect closure using clips and an endoloop for gastric subepithelial tumors arising from the muscularis propria. *Surg Endosc* 2014; **28**: 1978-83.

9 Inoue H, Tanabe M, Shimamura Y *et al.* A novel endoscopic purse-string suture technique, "loop 9", for gastrointestinal defect closure: A pilot study. *Endoscopy* 2022; **54**: 158-62.

10 Kobara H, Nishiyama N, Fujihara S *et al.* Traction-assisted endoscopic full-thickness resection followed by o-ring and over-the-scope clip closure in the stomach: An animal experimental study. *Endosc Int Open* 2021; **9**: E51-7.

11 Zhang Q, Jin HY, Shen ZH *et al.* Novel through-the-scope twin clip for the closure of gi wounds: The first experimental survival study in pigs (with videos). *Gastrointest Endosc* 2021; **94**: 850-8.e2.

12 Nomura T, Sugimoto S, Nakamura H *et al.* Reopenable clip over line method for the closure of full-thickness defect after gastric endoscopic full-thickness resection. *Endoscopy* Published on line: 6 May 2022; DOI: 10.1055/a-1824-4919.

13 Nomura T, Sugimoto S, Temma T, Oyamada J, Ito K, Kamei A. Reopenable clip-over-the-line method for closing large mucosal defects following gastric endoscopic submucosal dissection: Prospective feasibility study. *Dig Endosc* Published on line: 8 Nov 2022; DOI: 10.1111/den.14466.

14 Hu JW, Ge L, Zhou PH *et al.* A novel grasp-and-loop closure method for defect closure after endoscopic full-thickness resection (with video). *Surg Endosc* 2017; **31**: 4275-82.
